# Exit strategies following percutaneous nephrolithotomy (PCNL): a comparison of surgical outcomes in the Clinical Research Office of the Endourological Society (CROES) PCNL Global Study

**DOI:** 10.1007/s00345-012-0898-x

**Published:** 2012-07-01

**Authors:** Luigi Cormio, Gaspar Ibarlucea Gonzalez, David Tolley, Mario Sofer, Ahmet Muslumanoglu, Hans-Christoph Klingler, Jens-Uwe Stolzenburg, Jean de la Rosette

**Affiliations:** 1 Department of Urology, University of Foggia, Foggia, Italy; 2 Department of Urology, Hospital Galdakao-Usansolo, Bizkaia, Spain; 3 The Scottish Lithotriptor Centre, Western General Hospital, Edinburgh, Scotland, UK; 4 Department of Urology, Tel-Aviv Sourasky Medical Center, Tel-Aviv University, Tel Aviv, Israel; 5 Department of Urology, Haseki Training and Research Hospital, Istanbul, Turkey; 6 Department of Urology, Medical University of Vienna, Vienna, Austria; 7 Department of Urology, University of Leipzig, Leipzig, Germany; 8 Department of Urology, AMC University Hospital, Maibergdreef 9, 1105 AZ Amsterdam, The Netherlands

**Keywords:** Nephrostomy tube, Stent, PCNL, Tubeless, Urinary stones

## Abstract

**Purpose:**

To compare the characteristics and outcomes of exit strategies following percutaneous nephrolithotomy (PCNL) using the Clinical Research Office of the Endourological Society (CROES) PCNL Global Study database.

**Materials and methods:**

Two matched data sets were prepared in order to compare stent only versus NT only and TTL versus NT only. Patients were matched on the exit strategy using the following variables: case volume of the center where they underwent PCNL, stone burden, the presence of staghorn stone, size of sheath used at percutaneous access, the presence of bleeding during surgery, and treatment success status. For categorical variables, percentages were calculated and differences between the four groups were tested by the chi-square test.

**Results:**

The only significant difference reported between the matched pairs was between NT and stent only groups. NT only PCNL was associated with significantly longer operating times (*p* = 0.029) and longer hospital stay (*p* < 0.001) than stent only PCNL.

**Conclusions:**

Patients who undergo PCNL with less invasive exit strategy involving a stent only have shorter hospital stay than those who have postoperative NT. The intraoperative course is the primary driver of complications in PCNL and not necessarily the exit strategy.

## Introduction

Percutaneous nephrolithotomy (PCNL) is the recommended treatment option for large or otherwise complex renal or proximal ureteral stones [1]. The standard PCNL procedure involves creating a narrow percutaneous access to the kidney and the formation of a working tract connecting the flank surface with the intrarenal collecting system through which nephroscopy is performed. This allows endoscopic stone disintegration and removal of the stone fragments. A temporary nephrostomy tube (NT) is usually left in place at the end of the procedure to allow urinary drainage, tamponade of tract bleeding, and to maintain access to the collecting system should delayed “second-look” nephroscopy be necessary.

The practice of routine NT placement is, however, open to debate since 1997, when Bellman et al. [2] first demonstrated that a “tubeless” PCNL, whereby the NT was replaced by a double-J stent, was associated with less postoperative pain, less analgesia requirement, shorter hospital stay, and faster return to normal activities. Several randomized controlled trials (RCTs) and their meta-analyses [3, 4] suggest that the tubeless approach reduces postoperative pain and hospital stay and that substituting double-J stents with external ureteral catheters or no drainage at all [totally tubeless (TTL)] further improves patients’ compliance by eliminating stent-related symptoms and need for cystoscopic removal [5, 6]. On the other hand, other well-designed RCTs demonstrate advantages of early NT removal [7] or placement of small-bore NTs [8] over the tubeless approach. As a consequence, the optimal exit strategy after PCNL remains controversial.

The Clinical Research Office of the Endourological Society (CROES) conducted a prospective observational study collecting data of consecutive patients treated with PCNL over a 1-year period at the 96 participating centers around the world. The purpose of the CROES PCNL Global Study was to establish a prospective global database for the current indications and outcomes of PCNL. The analysis of the database was intended to facilitate better understanding of the fundamental differences between clinical institutions around the world in the use of this procedure and to identify specific factors that might influence treatment-related morbidity. The overall results for indications, complications, and outcomes in the cohort of over 5,800 patients treated at the centers participating in the CROES PCNL Global Study have already been reported [9, 10]. The present analysis aimed to provide a photograph of worldwide clinical practice with PCNL exit strategy and to compare the characteristics and outcomes of the different exit strategies adopted by centers participating at the CROES PCNL Global Study.

## Materials and methods

The organization and methods of the CROES PCNL Global Study have been described previously [9]. Patients were treated with PCNL during a 1-year period between November 2007 and December 2009. PCNL was carried out either in the supine or in the prone position. Access to the upper tract was guided by ultrasound and/or X-ray in combination with retrograde intrarenal contrast injection. Once access was obtained, a guidewire was inserted and preferably maneuvered toward the ureter. Dilation was performed with balloon, telescopic or serial dilators and an Amplatz sheath was then positioned. The collecting system was then inspected by nephroscope and the stones were either disintegrated by laser, ultrasound or ballistic devices or removed in toto with graspers. The procedure was considered to have completed when all removable stones had been taken out. Internal and/or and external drain(s) were positioned according to the judgment of the surgeon.

The need for transfusion was based on the clinical judgment of the treating physician and local clinical practice guidelines. Assessment of immediate stone clearance was performed by ultrasound, X-ray or computed tomography (CT) scanning, based on availability or local clinical practice. Perioperative complications were assessed and graded according to the modified Clavien System [11] as applied to PCNL [12]. Patients’ characteristics, surgical procedure and outcome data were analyzed according to the exit strategy, namely placement of NT without ureteral stenting (NT only); ureteral stenting without NT (stent only), and totally tubeless PCNL (TTL).

### Statistical analysis

Two matched data sets were prepared in order to compare stent only versus NT only and TTL versus NT only. The matched data sets were created using propensity score matching, a multidimensional matching technique based on multivariate logistic regression. Patients were matched on the exit strategy using the following variables: case volume of the center where they underwent PCNL, stone burden, the presence of staghorn stone, size of sheath used at percutaneous access, the presence of bleeding during surgery, and treatment success status. These matching factors were selected from a pool of preoperative characteristics that would determine the surgeon’s choice for exit strategy. For categorical variables, percentages were calculated and differences between the four groups were tested by the chi-square test with a level of significance of *p* < 0.05.

Contributions of individual investigators to the preparation of the manuscript and the significance of input in data collection were considered in authorship allocation according to the guidelines of CROES publications [13].

## Results

The characteristics of patients included in the matched pair analysis are shown in Table 1. In all groups, there were more males than females, patients were on average overweight, and the majority of patients had an American Society of Anesthesiologists (ASA) score of 1 or 2. The only significant difference between groups was that patients who received a NT only were more likely to have had previous open renal surgery than patients receiving a stent only.

**Table 1:** Patient characteristics according to exit procedure

	NT only *n* = 244	Stent only *n* = 244	*p* value	NT only *n* = 68	TTL *n* = 68	*p* value
Case volume [mean (SD)]	83.5 (66.1)	70.0 (59.5)		70.6 (48.9)	74.4 (59.7)	
Gender no. (%)						
Male	135 (55.6)	159 (65.2)	0.030	40 (58.8)	46 (67.6)	0.285
Female	108 (44.4)	159 (34.8)		28 (41.2)	22 (32.4)	
Age (years) [mean (range)]	49.5 (14.7)	49.4 (15.3)	0.929	47.4 (14.9)	48.2 (14.5)	0.748
BMI [mean (SD)]	26.9 (4.8)	26.4 (4.8)	0.384	26.8 (5.1)	26.5 (3.7)	0.620
ASA physical status classification [no. (%)]						
I	124 (51.0)	125 (53.6)	0.610	45 (68.2)	37 (55.2)	0.281
II	88 (36.2)	72 (30.9)		15 (22.7)	23 (34.3)	
III	27 (11.1)	32 (13.7)		6 (9.1)	7 (10.4)	
IV	4 (1.6)	4 (1.7)		0 (0.0)	0 (0.0)	
Antiplatelet/coagulant therapy no. (%)	19 (7.8)	17 (7.0)	0.729	2 (2.9)	5 (7.4)	0.437
Previous open renal surgery no. (%)	20 (8.2)	8 (3.3)	0.020	4 (5.9)	4 (5.9)	0.715
Renal anomalies no. (%)						
Ectopic	2 (0.8)	0 (0.0)	0.070	0 (0.0)	1 (1.5)	0.572
Horseshoe	9 (3.7)	2 (0.8)		1 (1.5)	1 (1.5)	
Malrotation	3 (1.2)	5 (2.0)		1 (1.5)	0 (0.0)	
Single kidney	9 (3.7)	3 (1.2)	0.143	1 (1.5)	1 (1.5)	1.000
Stone characteristics^a^						
Staghorn no. (%)	43 (17.6)	50 (20.5)	0.419	7 (10.3)	7 (10.3)	0.777
Multiple stones no. (%)	93 (38.1)	101 (44.4)	0.153	32 (52.9)	29 (57.4)	0.604
Single stone no. (%)	151 (61.9)	143 (58.6)		36 (47.1)	39 (42.6)	
Stone size (mm^3^) [mean (SD)]	330.0 (228.6)	301.4 (214.8)	0.559	333.2 (189.7)	295.3 (186.7)	0.717

*ASA* American society of anesthesiologists, *BMI* body mass index, *NT* nephrostomy tube, *TTL* totally tubeless

^a^Analysis was done on complete data sets. Missing observations were excluded

**Fig. 1:**
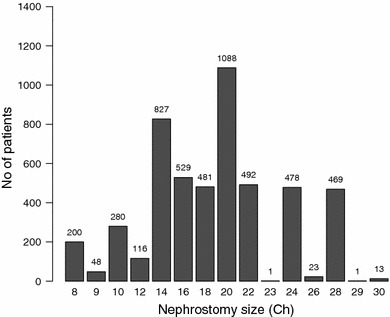
Distribution of patients according to the nephrostomy size

The distribution of patients according to the size of the NT is shown in Fig. 1. Data were available for 5,046 patients. The most commonly sized NT used was a 20 Ch (21.6 %) followed by a 14 Ch NT (16.4 %). In terms of operative procedure, the only significant difference between groups reported was between the NT and stent only groups in regard to percutaneous access point. No differences were observed between groups in stone-free rates and the incidence of bleeding (Table 2). Mean duration of PCNL across treatment groups ranged from 67 to 82 min (Table 3). Mean operating time was significantly longer for patients who had NT only compared with patients who had ST only (*p* = 0.029). Postoperative hospital stay was also significantly longer for NT only compared with ST only patients (*p* < 0.001). No other significant differences between the two matched groups were reported.

**Table 2:** Operative characteristics according to exit procedure

	NT only *n* = 244	Stent only *n* = 244	*p* value	NT only *n* = 68	TTL *n* = 68	*p* value
Position						
Supine no. (%)	36 (14.8)	50 (20.5)	0.096	19 (27.9)	15 (22.1)	0.428
Prone no. (%)	208 (85.2)	194 (79.5)		49 (72.1)	53 (77.9)	
Percutaneous access						
Lower calyx no. (%)	171 (70.4)	129 (53.1)	0.001	61 (89.7)	50 (73.5)	0.080
Middle calyx no. (%)	39 (16.0)	63 (25.9)		6 (8.8)	12 (17.6)	
Upper calyx no. (%)	19 (7.8)	47 (19.3)		1 (1.5)	5 (7.4)	
Multiple calyces no. (%)	14 (5.8)	4 (1.6)		0 (0.0)	1 (1.5)	
Tract dilation						
Telescopic dilator no. (%)	119 (48.8)	135 (55.3)	0.147	21 (69.1)	28 (58.8)	0.211
Balloon dilator no. (%)	125 (51.2)	109 (44.7)		47 (30.9)	40 (41.2)	
Postoperative stone-free rate no. (%)	213 (87.3)	217 (88.9)	0.575	62 (91.2)	62 (91.2)	1.00
Reported bleeding no. (%)	8 (3.3)	6 (2.5)	0.587	2 (2.9)	2 (2.9)	1.00

*NT* nephrostomy tube, *TTL* totally tubeless

**Table 3:** Outcome of the exit procedures

	NT only *n* = 244	Stent only *n* = 244	*p* value	NT only *n* = 68	TTL *n* = 68	*p* value
Operative time (min) [mean (SD)]	81.5 (44.7)	73.2 (37.1)	0.029	67.1 (39.1)	77.6 (49.7)	0.181
Hemoglobin decrease (g/dL) [mean (SD)]	3.8 (3.1)	3.5 (3.5)	0.327	3.4 (2.8)	3.2 (3.3)	0.746
Fever no. (%)	22 (9.1)	20 (8.3)	0.757	3 (4.4)	4 (5.9)	0.697
Clavien graded complications no. (%)						
I	12 (5.0)	16 (6.7)	0.555	5 (7.5)	2 (2.9)	0.594
II	11 (4.6)	14 (5.9)		1 (1.5)	1 (1.5)	
IIIa	2 (0.8)	5 (2.1)		0 (0.0)	1 (1.5)	
IIIb	1 (0.4)	2 (0.8)		1 (1.5)	2 (2.9)	
IVa	0 (0.0)	1 (0.4)		0 (0.0)	1 (1.5)	
IVb	0 (0.0)	0 (0.0)		0 (0.0)	1 (1.5)	
V	0 (0.0)	0 (0.0)		0 (0.0)	0 (0.0)	
Postoperative hospital stay (days) [mean (SD)]	4.3 (3.2)	2.3 (1.9)	<0.001	4.0 (2.9)	3.2 (3.4)	0.107

*NT* nephrostomy tube, *TTL* totally tubeless

## Discussion

In the past decade, there has been continuing interest in the concept of foregoing NT placement after PCNL with the intent of reducing some postoperative problems associated with this policy, such as patient’s discomfort, urinary leakage from the percutaneous tract, and prolonged hospital stay. Based on several RCTs demonstrating efficacy and safety of tubeless PCNL, such approach is currently recommended in the European Association of Urology guidelines [1] as a safe alternative to NT placement in uncomplicated cases. Reasons for placing a NT at the end of PCNL include bleeding from the tract requiring tamponade, keeping an access for a “second-look” procedure when stone clearance is considered incomplete, and providing urinary drainage, though this could be achieved by a ureteral catheter or a double-J stent.

Several RCTs and their meta-analyses [3, 4] have shown that tubeless PCNL provides less postoperative pain, less postoperative urinary leakage and shorter hospital stay than NT placement. A report by Cormio et al. on the use of TachoSil^®^ has been published recently [14]. Results showed that compared with NT placement, complication rates were lower, including urinary leakage, and hospital stay was shorter. Pain and analgesic use were similar with the two procedures. In the present study, which provides a photograph of real-life clinical practice worldwide, no differences were reported between a matched pair analysis of tubeless PCNL and stent only placement. The patients were matched for clinical characteristics so removing any selection bias. These findings remain interesting in view of the fact that tubeless PCNL was applied also to some “complicated cases”. Accordingly, TTL PCNL has been shown to be safe and effective in cases of complex renal stone disease [15, 16] and even in cases complicated with hemorrhage [17]. Similarly, TTL PCNL has been found to be safe and effective also in cases of moderate to large stone burden [18], renal anomalies [19], and elderly patients [20].

In the present study, operating time and postoperative hospital stay were both significantly shorter for patients receiving the less invasive stent only exit compared with the more invasive NT. This observation remained valid after matching the patient groups based on the patients’ characteristics that would predispose a surgeon to choose one exit strategy instead of the other. Our results therefore confirm that shorter hospital stay is indeed an advantage of less invasive exit strategies.

This analysis did not reveal statistical differences in complications between patients who received stents versus NT or TTL versus NT recipients. This finding suggests that the preoperative characteristics of the patients and the clinical course of the surgery are the main drivers of complication rate when considering the type of exit strategy. Matched comparisons resulted into comparable groups of patient with rather similar preoperative characteristics and intraoperative course.

## Conclusions

Patients who undergo PCNL with less invasive exit strategy involving a stent only have shorter hospital stay than those who have postoperative NT. The intraoperative course is the primary driver of complications in PCNL and not necessarily the exit strategy. Consequently, the choice of exit strategy should be based on intraoperative course of the PCNL.
